# Alternative air–liquid interface method for inhalation toxicity testing of a petroleum-derived substance

**DOI:** 10.1016/j.mex.2020.101088

**Published:** 2020-10-08

**Authors:** Verstraelen Sandra, Jacobs An, Van Laer Jo, Van Deun Masha, Bertels Diane, Hilda Witters, Remy Sylvie, Geerts Lieve, Deferme Lize, Frijns Evelien

**Affiliations:** a VITO NV (Flemish Institute for Technological Research), Unit HEALTH, Mol, Belgium; b VITO NV, Unit SCT (Separation and Conversion Technology), Mol, Belgium; c Department of Biomedical Sciences, University of Antwerp, Antwerp, Belgium; d ExxonMobil Petroleum and Chemical B.V., Machelen, Belgium

**Keywords:** *In vitro* inhalation testing, Passive dosing, Gasoline

## Abstract

*In vitro*-based new approach methodologies (NAMs) provide a pragmatic solution to animal testing of petroleum substances and their constituents. A previous study exposed an in vitro model (A549 cells) at the air–liquid interface (ALI) to assess inhalation toxicity of a single compound, ethylbenzene. Experimental conditions using VITROCELL^Ⓡ^ 24/48 exposure system were optimized to achieve a deposition efficiency that resulted in dose-dependent biological changes. The feasibility of this set-up was evaluated for testing the complex substance gasoline, which, at only high concentrations, can induce mild respiratory irritation in animals and cough in humans.

• Results showed that perpendicular ALI exposure flow systems (VITROCELL® 6/4 and 24/48) may not be appropriate for testing gasoline because it was not possible to achieve enough deposition onto the cells and in the culture medium to measure dose and to determine dose-dependent biological changes (more information can be found in ‘Supplementary material and/or Additional information’ section).
• Structural features (*e.g.* aromatic or saturated hydrocarbon structure) and high hydrophobicity, together with the low concentrations of individual components in gasoline, may have caused the low deposition.
• To achieve a higher deposition on the cells, A549 cells were exposed to gasoline at the ALI by passive dosing.

The results demonstrate that the presented methodology is a promising NAM for inhalation toxicity testing of (semi-)volatile complex substances with low aqueous solubility.

## Graphical abstract

**Figure:**
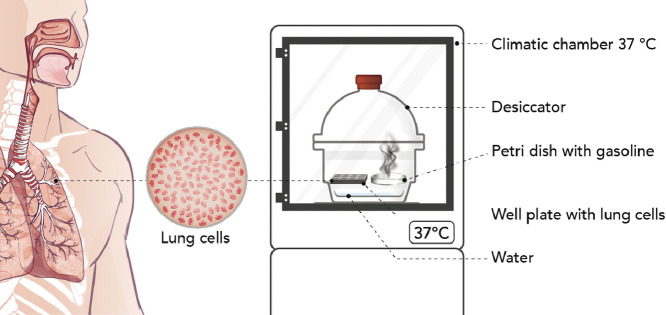

Specifications Table

**Table:** 

Subject Area:	Pharmacology, Toxicology and Pharmaceutical Science
More specific subject area:	*In Vitro* Inhalation Toxicology
Method name:	Air–Liquid Interface Passive Dosing Inhalation Exposure
Name and reference of original method:	Optimization and validation of an *in vitro* air–liquid interface acute inhalation testing system for petroleum substances and it constituents, ready for submission to a relevant Journal.
Resource availability:	Not applicable

## A549 lung cell model and culture conditions

The human alveolar epithelial type 2-like A549 cell line was obtained from American Type Culture Collection (ATCC number: CCL-185, Manassas, USA) and was originally derived from a lung carcinomatous tissue from a 58-year-old Caucasian male. A549 cells were grown in T-75 culture flasks (Greiner Bio-One, Vilvoorde, Belgium) and routinely maintained in Minimal Essential Medium (MEM) 1x with GlutaMAX™-1 (Brand Gibco, ThermoFisher Scientific, Waltham, Massachusetts, USA) supplemented with 10% non-heat inactivated fetal bovine serum (FBS superior, Merck, Darmstadt, Germany) at 37 °C under 5% carbon dioxide (CO_2_). Before reaching 70–80% confluence, cells were sub-cultured using (0.05%) Trypsin-EDTA solution (Brand Gibco, ThermoFisher Scientific). Medium was refreshed every 2 days and cells were sub-cultured every 3 (9 × 10^5^ cells in 20 ml cell culture medium (CCM)) or 4 days (4.5 × 10^5^ cells in 20 ml CCM). Cells were passaged at least twice before use in experiments and no more than 20 times in total.

## *In vitro* air–liquid interface passive dosing exposure

A549 cells were seeded at a density of 151,515 cells/cm² (50,000 cells/insert) on ThinCert™ polystyrene membrane inserts, pore size 0.4 µm, surface 0.33 cm² (Greiner Bio-One, catalog number 662 641). Inserts were placed in a sterile 24-well plate (Greiner Bio-One, catalog number 662 160), and CCM was added to both sides, 600 µl basolateral and 100 µl apical side. Plates were incubated for ± 72 h (h) at 37 °C, 5% CO_2_ in a humidified incubator.

Immediately before exposure, CCM was completely removed from the apical side and the inserts were transferred in a stainless steel 24-well plate into a humidified desiccator (150 DN VWR#467-0070, filled with 500 ml water at the bottom), which was placed in a climatic chamber at 37 °C. Before positioning the inserts in the plate, each well was separately filled with CCM (600 µl) allowing cells to be nourished from the bottom while being exposed via the top side for 1 h.^1^

**Fig. 1:**
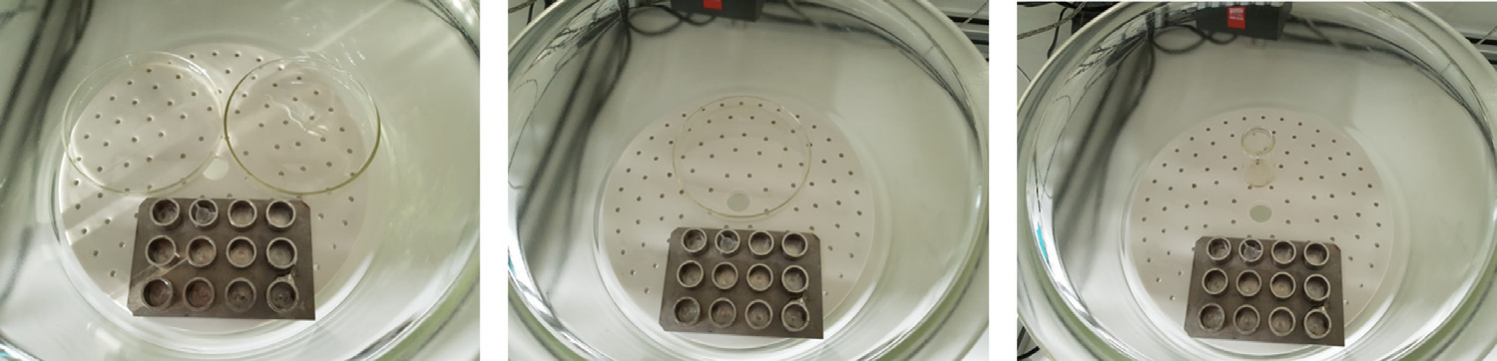
Passive exposure of A549 cells using 2 Petri dishes filled with 6 ml gasoline each in a humidified desiccator in a climatic chamber at 37 °C.

In the desiccator, A549 cells were passively exposed to the surrounding ‘clean’ air (CA, #4 inserts) *versus* 3 arbitrary chosen conditions of gasoline: 1 glass vial (Ø20 mm) with 6 ml gasoline, 1 Petri dish (Ø110 mm) with 6 ml gasoline, and 2 Petri dishes (Ø110 mm) with 6 ml of gasoline (Fig. 1) (#6 inserts/condition; 3 for cell viability determination and 3 for chemical analysis). Incubator control (IC) cells, consisted of cell culture inserts (#4 inserts) without apical medium, were kept in a humidified 37 °C incubator with 5% CO_2_ for 1 h and served as control for passively CA exposure.

For air–liquid interface (ALI) post-incubation, the inserts were placed in a new sterile 24-well plate with 600 µl growth medium basolateral and allowed recovery period of +/- 23 h in a humidified 37 °C incubator with 5% CO_2_ for further assessment of cell viability (MTT assay). Three biologically independent runs, using different cell passages, were performed. The technical details are shown in Table 1. All materials (*e.g.,* stainless steel plate, desiccator) were cleaned using 70% ethanol after use.

**Table 1:** Technical details passive dosing of A549 cells using gasoline.

*Passive exposure system*	Desiccator in climatic chamber (37 °C)
*Respiratory cell model*	A549
*Type of inserts*	ThinCert™ polystyrene membrane inserts (Greiner Bio-One), pore size 0.4 µm, surface 0.33 cm^2^ (24-well)
*Seeding density on inserts*	50,000 cells/insert or 151,515 cells/cm^2^
*Growth protocol*	72 h (h) submerged growth before ALI exposure
*Conditioning*	37 °C and 100% relative humidity, 500 ml below ceramic plate
*Concentration-range*	Incubator control (**IC**): no apical medium, 24 h in incubator, control for ‘clean’ air (CA); *n* = 4
	**CA**; *n* = 4
	Gasoline: 1 open glass vial (φ20 mm) with 6 ml, 1 open Petri dish (φ100 mm) with 6 ml, 2 open Petri dishes (φ100 mm) with 6 ml each; *n* = 6
*Exposure time*	1 h
*Post-incubation time*	ALI; +/- 23 h
*Endpoints*	Cell viability (MTT); after 23 h post-incubation time
*Chemical analysis generated dose*	Sorbent tubes, gas chromatography-mass spectrometry (GC–MS); in desiccator headspace
*Chemical analysis deposited dose*	Headspace (HS)-GC–MS, total gasoline and BTEX (benzene, toluene, ethylbenzene, and (p + m)-xylene, o-xylene), in cells/cell culture medium (CCM)
*Independent biological experiments*	3

## Chemical analysis in desiccator headspace (generated dose)

**Fig. 2:**
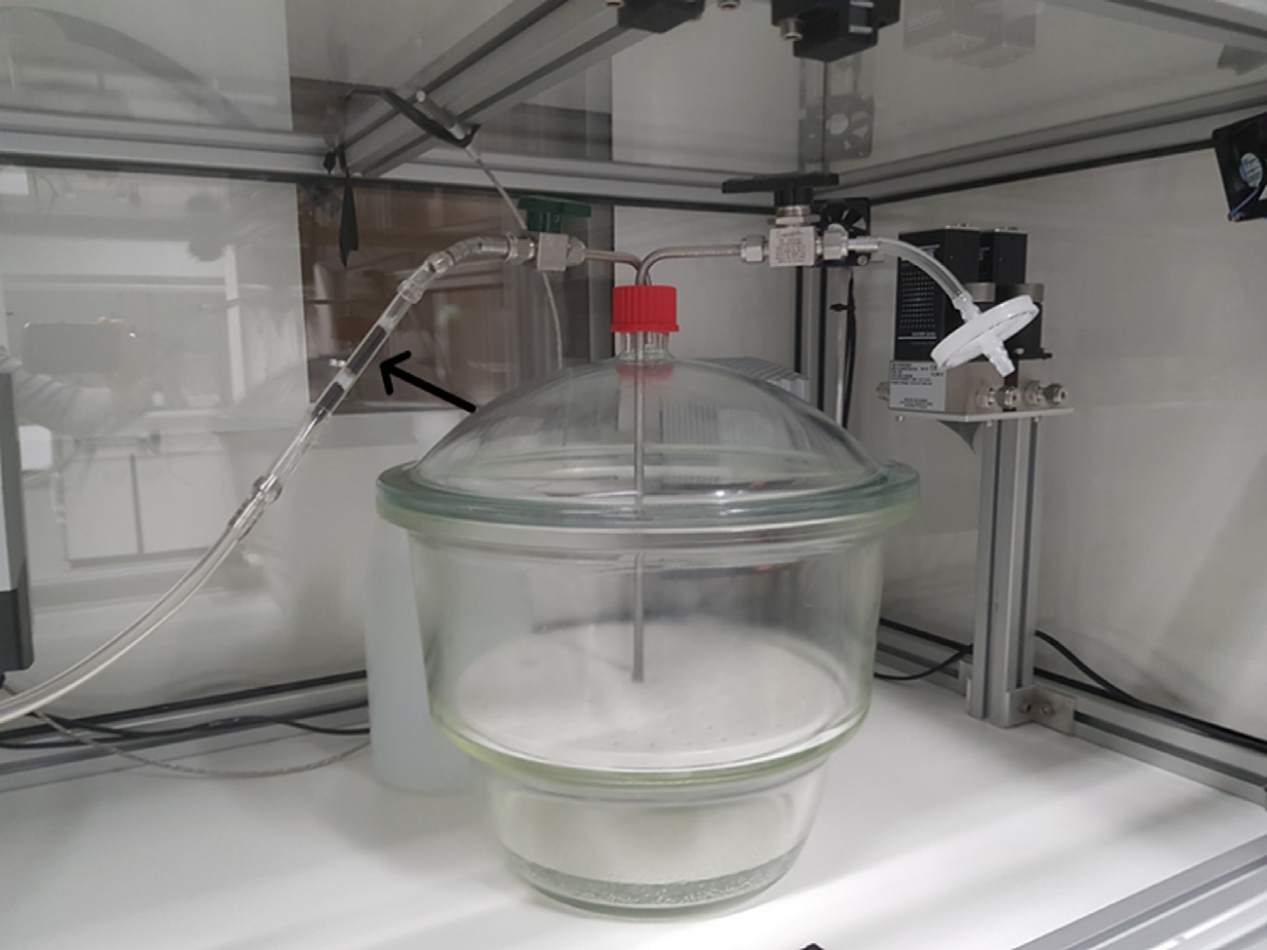
Set up for sorbent tube (arrow) sampling of desiccator headspace.

The generated concentration of gasoline in the desiccator headspace was determined after 1 h exposure by sampling an air volume of 10 mL per min (mlpm) for 10 min (min) through a sampling tube filled with coconut activated charcoal (SKC 226–09) (Fig. 2). The generated concentration gasoline was determined for each exposure condition (glass vial (Ø20 mm), 1 Petri dish (Ø110 mm), and 2 Petri dishes (Ø110 mm)). During sampling, a HEPA filtered inlet was opened to prevent vacuum.

The sorbent tubes were extracted from the activated charcoal by using chemical extraction using a carbon disulphide solution with an internal standard (2-fluorotoluene). The extract was then analysed using an Agilent 6890 N GC coupled to an Agilent 5975 MS (Agilent technologies, US).

The separation of the individual volatile organic compounds (VOC) was carried out on a non-polar GC column (Rtx-502.2, 30 m; 0.25 mm id; df 1.4 µm) using helium as carrier gas. To quantify gasoline linear regression with an internal standard method was used. The air concentration was calculated based on the amount in the extract, the sample rate, and the sampling time.

## Chemical analysis (deposited dose)

### Deposited dose in/on cells and cell culture medium

For chemical analysis, directly after exposure of A549 cells, 100 µl medium was added to the apical side and cells were scrapped from the membrane. After scrapping, the 100 µl medium containing the cells was added to a 20 ml glass vial which contained 4.5 ml of blanc water. The vials were immediately airtight sealed with aluminum caps. For comparison also the membranes were cut from the inserts and added to similar glass with blank water and sealed airtight for headspace-gas chromatography-mass spectrometry (HS-GC–MS) analysis. From each gasoline condition, 600 µl of CCM was collected in a glass vial which contained 4.5 ml blank water for HS-GC–MS analysis.

For the measurement of total gasoline and BTEX (benzene, toluene, ethylbenzene, and (p + m)-xylene, o-xylene) the samples (cells and/or CCM dispersed in blank water) were doped with the isotope-labelled compound D10-EB by injection through the membrane in the lid of the sealed vial. The HS sampler heats the vial at 70 °C for 30 min. During this period the gasoline transitions from the sample matrix into the vapor phase above. A fixed volume of the headspace vapor is extracted from the vial and injected into a capillary column for GC separation. A MS is used to detect and quantify the gasoline or BTEX (Thermo HS-GC–MS).

For the measurement of total gasoline scan mode (mass 30 to 250) was used. The internal standard method is used for the quantitative determination of gasoline. The quantification is based on the integrated peak area of the total ion current chromatogram of the gasoline (RT 3 min to 30 min) and the most characteristic ion for D10-EB. A calibration standard of gasoline and internal standard was used to determine the response factor and calculate the concentration.

The total gasoline scan mode showed distinct peaks of BTEX in the chromatogram. For this reason selective ion monitoring (SIM) was performed focusing on these ions.

For the measurement of BTEX SIM mode was used to increase sensitivity. The internal standard method was also used for the quantitative determination of BTEX. The quantification is based on the integrated peak area of the most characteristic ion for all these components and for the internal standard. A calibration standard of benzene, toluene, ethylbenzene, and (p + m)-xylene, o-xylene and D10-EB is used to determine the response factor and calculate the concentrations.

### Deposited dose in stainless steel inserts (without cells)

**Fig. 3:**
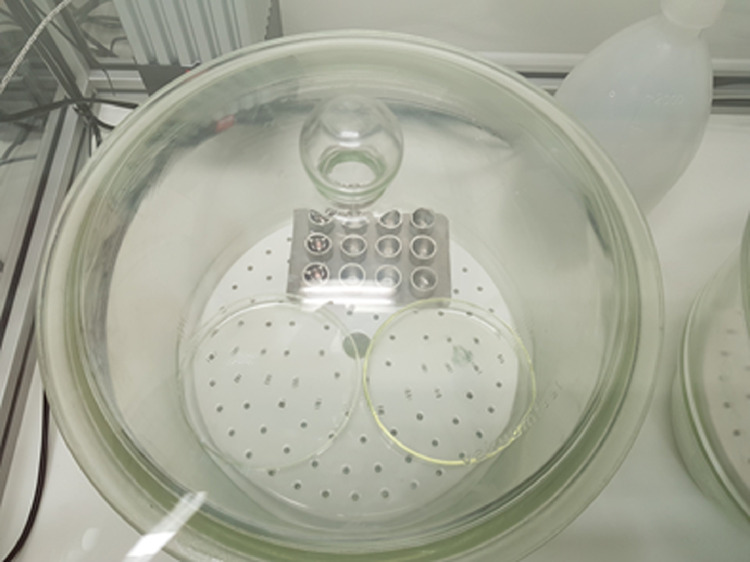
Stainless steel inserts in stainless steel well plate in desiccator in climatic chamber for deposited dose determination.

The deposited dose was also determined in 24-well stainless steel inserts without cells. The inserts were positioned in the stainless steel well plate in the desiccator (Fig. 3). CCM (600 µl) was added to the basolateral side and 125 µl was pipetted inside the stainless steel inserts (apical). Three inserts were used for each exposure condition (glass vial, Petri dish, and 2 Petri dishes). After exposure, the CCM from the apical and basolateral side was pipetted in the vials with 4,5 ml blank water and sealed for HS-GC–MS analysis. The HS-GC–MS measurement method is described under deposited dose determination in/on cells/CCM.

After experimental work, the stainless steel plate was cleaned using 70% ethanol.

## Cell viability determination

To assess cell viability, several assays are available for application in an ALI set-up. Here, an MTT assay (Brand Acros Organics, ThermoFisher Scientific, catalog number 158,990,010) was performed to measure mitochondrial activity. The conversion of MTT (3-(4,5-dimethylthiazol-2-yl)−2,5-diphenyltetrazolium bromide) tetrazolium salt into its reduced formazan form was assessed. A MTT stock was prepared in Dulbecco's Phosphate Buffered saline at a concentration of 5 mg/ml. The MTT substrate is prepared in CCM and added to cells in culture, at a final concentration of 1 mg/ml, and incubated for 2–3 h at 37 °C and 5% CO_2_. The formazan product of the MTT tetrazolium accumulates as an insoluble precipitate inside cells as well as being deposited near the cell surface and in the CCM. The formazan must be solubilized prior to recording absorbance readings by *e.g.,* isopropanol (2 h incubation, shaking at room temperature). The quantity of formazan (presumably directly proportional to the number of viable cells) is measured by recording changes using a multi-mode microplate reader in absorbance mode (570 nm; Clariostar, BMG Labtech, Offenburg, Germany). Results were expressed as percentages of non-treated negative control (CA) cells. Significant changes in cell viability (MTT) were analysed relative to CA and were assessed by mixed models while considering experiment ID (biological replicate) as random factor. Data were analysed using R [1] and specific packages for mixed model analyses “lme4” [2] and “lmerTest” [3]. P-value smaller than 0.05 was used as cut-off for statistical significance.

## Method validation

### Generated dose

The generated concentration gasoline in the desiccator headspace was determined by sampling air from the headspace through a sorbent tube filled with coconut activated charcoal [4]. For each exposure condition (glass vial, Petri dish, and 2 Petri dishes) one sorbent tube was sampled. The results of the chemical analysis are shown in Table 2. Highest Total Volatile Organic Compounds (TVOC) concentrations in the desiccator headspace of 875.100 mg/m^3^ was measured for the exposure condition with two Petri dishes. The individual VOCs with highest concentrations were toluene, hexane and pentane, corresponding respectively with 17.03, 1.4, and 6.12% in gasoline sample.

**Table 2:** Concentration of individual and total volatile organic compounds (TVOC) (mg/m^3^) in the headspace of the desiccator for 3 exposure conditions (glass vial, Petri dish, 2 Petri dishes).

	6 ml glass vial (mg/m³)	6 ml Petri dish (mg/m³)	2 × 6 ml Petri dish (mg/m³)
Pentane	36.400	59.500	109.400
Trans-1,2-dichloorethene	<	35	52
Hexane	8.600	61.000	110.400
Cyclohexane	970	9.100	16.300
Heptane	1.900	27.600	49.900
**Benzene**	3.100	24.600	44.200
n-Octane	254	3.600	6.600
**Toluene**	13.700	155.200	283.200
n-Nonane	<	253	520
**Ethylbenzene**	790	6.600	13.900
**m** **+** **p-Xylene**	2.380	19.800	43.200
**o-Xylene**	650	4.700	10.900
Cumene	<	141	330
1,3,5-Trimethylbenzene	<	73	276
1,2,4-Trimethylbenzene	62	255	930
**TVOC**	**86.200**	**565.800**	**875.100**

### Deposited dose in/on cells and in cell culture medium

The deposited dose was determined in/on cells and in CCM using HS-GC–MS. Three independent biological experiments were run for each exposure condition (glass vial, Petri dish, and 2 Petri dishes). The highest signals were measured for BTEX. For that reason, BTEX results are shown here. It can be concluded that high BTEX concentrations were measured and the concentrations in CCM were much higher than those measured in/on the cells (Table 3).

**Table 3:** Average (Avg) and standard deviation (SD) of BTEX (benzene, toluene, ethylbenzene, and (p + m)-xylene, o-xylene) concentration in/on cells (apical) and in cell culture medium (CCM, basolateral) per exposure condition for 3 independent biological experiments.

		Avg +/- SD of BTEX (mg)	Avg +/- SD of BTEX (mg/cm^2^)
**6** **ml glass vial**	Apical	0.007 +/- 0.001	0.022 +/- 0.004
	Basolateral	1.1 +/- 0.5	3.2 +/- 1.4
6 ml Petri dish	Apical	0.4 +/- 0.3	1.3 +/- 1.0
	Basolateral	22.3 +/- 6.8	67.5 +/- 20.6
**2** **×** **6** **ml Petri dish**	Apical	1.3 +/- 0.7	4.0 +/- 2.1
	Basolateral	32.2 +/- 14.1	97.7 +/- 42.7

### Deposited dose in stainless steel inserts (without cells)

The deposited dose was determined in 24-well stainless steel inserts without cells. Three inserts were used for each exposure condition (glass vial, Petri dish, and 2 Petri dishes). After exposure, the CCM from the apical and basolateral side was used for HS-GC–MS analysis. This experiment was repeated 3 times. The highest signals were measured for BTEX. For that reason, BTEX results are shown here. It can be concluded that high BTEX concentrations were measured (Table 4).

**Table 4:** Average (Avg) and standard deviation (SD) of BTEX (benzene, toluene, ethylbenzene, and (p + m)-xylene, o-xylene) concentration inside insert (apical) and in medium (basolateral) per exposure condition for 3 runs.

		Avg +/- SD of BTEX (mg)	Avg +/- SD of BTEX (mg/cm^2^)
**6** **ml glass vial**	Apical	0.3 +/- 0.1	1.0 +/- 0.4
	Basolateral	1.2 +/- 0.5	3.6 +/- 1.5
**6** **ml Petri dish**	Apical	6.0 +/- 0.1	18.2 +/- 0.5
	Basolateral	21.8 +/- 1.2	66.1 +/- 3.5
**2** **×** **6** **ml Petri dish**	Apical	9.7 +/- 1.3	29.5 +/- 3.9
	Basolateral	30.9 +/- 5.0	93.7 +/- 15.1

### Cell viability

**Fig. 4:**
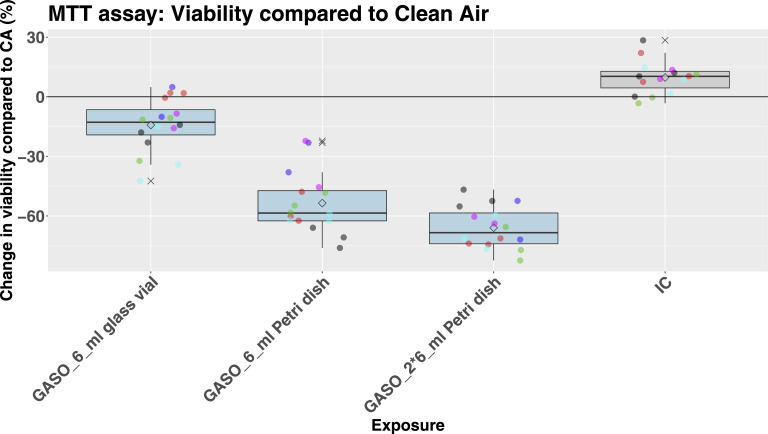
Change in cell viability (as % compared to CA) of A549 cells after 1 h passive exposure to gasoline (glass vial, 1 Petri dish, 2 Petri dishes, filled with 6 ml gasoline each) based on 5 runs (of which 3 independent experiments). Box and whisker plots visualizing the range of the individual data points per condition. The upper whisker extends from the hinge to the largest value no further than 1.5 * IQR from the hinge (where IQR is the inter-quartile range, or distance between the first and third quartiles). The lower whisker extends from the hinge to the smallest value at most 1.5 * IQR of the hinge. Data beyond the end of the whiskers are called "outlying" points and are plotted with an “x”; other individual data points are overlayed and plotted with filled dots. The mean is indicated by a square.

The mean cell viability (MTT) for A549 cells (3 independent runs, *N* = 3 + 2) was 89% after exposure to CA *versus* IC (*P* = 6.46E-03). Passive exposure of A549 cells to gasoline induced a concentration-dependent decreased mean cell viability of 86% (glass vial), 47% (1 Petri dish), and 34% (2 Petri dishes) respectively, as compared to CA. The results were statistically significant for the lower to higher tested concentrations, respectively *P* = 5.30E-4, *P* = 6.80E-22, and *P* = 7.00E-27. Results are shown in Fig. 4.

## Conclusion

The ultimate goal of this study was to use an ALI exposed *in vitro* model to assess the potential for inhalation toxicity of gasoline. It was found that active perpendicular ALI exposure flow systems (VITROCELL^Ⓡ^ 6/4 and 24/48) may not be appropriate for testing gasoline because it was not possible to achieve enough deposition onto the cells and in the CCM to measure the dose and to determine dose-dependent biological changes. Structural features (*e.g.,* aromatic structure or saturated hydrocarbon structure) and high hydrophobicity, together with the low concentrations of individual components in the gasoline, may have caused the low deposition, as was demonstrated by Steiner et al. with tobacco smoke (*e.g.,* for toluene the average delivery efficiency was 0.039%, for different smoke concentrations) [5]. For that reason, A549 cells were exposed to gasoline at the ALI by passive dosing, an approach that is already used in aquatic toxicity testing [6,7]. In this proof-of-concept study, we chose a worst case exposure to obtain an adverse and dose-dependent effect, and data that can support ‘Derived No Effect Level’ calculations. Gasoline is just one trial, many other petroleum substances do not have animal or human effect data for inhalation and extrapolation is done from other exposure data (mainly dermal) while there is certainly occupational exposure via the inhalation route so it is important to understand all possible hazards in controlled exposures but also during accidental exposure to possible extremely high concentrations (*i.e.,* spills, explosions, contaminations etc.).

On 3 independent experimental days, A549 cells were exposed to a concentration-range of gasoline (glass vial (86.200 mg/m^3^ TVOC), Petri dish (565.800 mg/m^3^ TVOC), and 2 Petri dishes (875.100 mg/m^3^ TVOC)), resulting in a significant concentration-dependent decrease in mean cell viability (MTT) of 86% (glass vial), 47% (1 Petri dish), and 34% (2 Petri dishes) as compared to the negative control. Deposited BTEX dose as proxy for gasoline dose was also determined using HS-GC–MS analysis. The dose in/on the cells was 0.022 +/- 0.004 mg/cm^2^ (glass vial), 1.3 +/- 1.0 mg/m^2^ (1 Petri Dish), and 4.0 +/- 2.1 mg/cm^2^ (2 Petri dishes). The BTEX dose in stainless steel inserts was found to be 47, 14, and 7-times higher than the dose measured in/on cells.

A549 cells exposed at the ALI to different gasoline concentrations by **passive dosing** showed a clear dose-dependent biological response. This NAM might be promising for inhalation toxicity testing of (semi-)volatile complex substances.

With these data, an alternative inhalation testing method based on passive dosing shows promising results for the complex petroleum-derived substance gasoline. Further improvements on the study design can be made, *e.g.,*: i) exposure to realistic *in vivo*-like concentrations to check for *in vitro* to *in vivo* extrapolations (IVIVE) since the high doses used in this study inducing respiratory toxicity do not reflect findings *in vivo*, ii) exposure to real-life concentrations (*e.g.,* worker exposure, 8 h) or repeated exposure, iii) determine advantages of using a 2D cell line (A549) *versus* a 3D human reconstructed tissue model or an alternative cell line (*e.g.,* BEAS-2B, Calu-3); use of a desiccator with a comparable human lung air volume (~4–6 l), iv) development of inserts with materials that are not affected by VOC (no use of polystyrene housing and polyethylene terephthalate membrane).

## Declaration of Competing Interest

The authors declare that they have no known competing financial interests or personal relationships that could have appeared to influence the work reported in this paper. [Include at least one reference, to the original publication of the method you customized.]

## Appendix Supplementary materials

Image, application 1

## Footnotes

1 One hour was the maximal exposure time which did not impair the polystyrene (PS) well plate and inserts (PS housing, polyethylene terephthalate (PET) membrane) by gasoline (maximal concentration 2 Petri dishes).
