# Supplementary material for: Alternative air–liquid interface method for inhalation toxicity testing of a petroleum-derived substance
Source: MethodsX. 2020 Oct 8;7:101088. doi: 10.1016/j.mex.2020.101088 (PMC7581970; doi:10.1016/j.mex.2020.101088)
Supplement: Supplementary file 1 [file mmc1.docx]

**Supplementary material *and/or* Additional information:**

A previous study successfully used an air-liquid interface (ALI) exposed *in vitro* model to mimic airway exposures of the single compound ethylbenzene (EB), often present in complex petroleum substances, in order to assess the potential for respiratory toxicity. Experimental conditions using **VITROCELL^®^ 24/48** exposure system were optimized to achieve a deposition efficiency that resulted in dose-dependent biological changes (1).

Based on these results using EB, an experimental set-up condition of 4 hours (h) gasoline aerosolization and deposition at ALI in the **VITROCELL^®^ 24/48** exposure system, followed by post-exposure incubation under ALI conditions in the cell incubator (20 h for cell viability/cytotoxicity and cytokine secretion, 1 h for gene expression) has been established. A549 cells were exposed to an average concentration-range of about 5600, 8400, and 11000 mg/m^3^ in 3 independent experimental runs. Generation up to a maximum of 11000 mg/m^3^ gasoline gave no effect on A549 cell viability (MTT assay). Additional endpoints, such as inflammation and oxidative stress were measured. Exposure of A549 cells to 11000 mg/m^3^ gasoline induced an increase of pro-inflammatory markers *interleukin* (*IL*)*6* (log2 fold change (FC) = 1.94, *P* = 7.28E-3), *IL8* (log2 FC = 1.98, *P* = 2.18E-3), and *C-C Motif Chemokine Ligand 2* (*CCL2*) (log2 FC = 1.13, *P* = 3.08E-3) as compared to clean air (CA), which was statistically significant for all markers. The oxidative stress marker *superoxide dismutase* (*SOD*)*2* showed a statistically significant increase for 11000 mg/m^3^ (log2 FC of ~ 0.56, borderline result). No gasoline was detected in/on the cells or in the cell culture medium (CCM) using headspace-gas chromatography-mass spectrometry (HS-GC-MS). As we know from the EB study, the deposition efficiency of the exposure system is low (<1%). Also, the compound-specific physicochemical properties (hydrophobic) will be a disadvantage for cell absorption. Gasoline is a complex petroleum substance with many constituents in low and variable concentrations. This characteristic, in combination with low deposition efficiency and its unfavourable physicochemical properties, resulted in a limited dose that could not result in biological effects.

Three possible options to increase the deposited dose were evaluated. The first option was to increase the generated concentration. However, generation of higher concentration levels of gasoline (>11 000 mg/m^3^) resulted in condensation in the tubing when lowering the temperature to 37 °C (maximum temperature for cell exposure). Condensation resulted in losses of heavier components and not all initial volatized gasoline reached the cells. The second possibility was to increase deposition efficiency, by *e.g.* using another ALI exposure system.

Since the **VITROCELL^®^ 6/4** module has a higher deposition efficiency for particles (~2%) compared to the VITROCELL^®^ 24/48 module (<1%), experiments were performed to check if gasoline (about 10000 mg/m^3^) was measured in/on the cells and in the CCM after exposure using HS-GC-MS. No gasoline was measured in/on the cells and in CCM. In addition, a worst-case exposure up to 50000 mg/m^3^ (with condensation in the tubing) was performed_._ Only a limited concentration of gasoline was measured in the CCM, but did not result in biological effects.

Both active perpendicular ALI exposure flow systems are not appropriate for testing gasoline because it was not possible to achieve enough deposition onto the cells and in the CCM to measure this dose and to determine dose-related biological changes. For that reason, we exposed A549 cells at the ALI to the complex substance gasoline by passive dosing, which showed promising results.

**References**

[1] Verstraelen S, Jacobs A, Van Laer J, Hollanders K, Van Deun M, Brabers R, Witters H, Remy S, Geerts L, Deferme L, Frijns E. Optimization and validation of an *in vitro* air-liquid interface acute inhalation testing system for petroleum substances and it constituents, ready for submission to a relevant Journal
